# Does arthroscopic sub-acromial decompression really work for sub-acromial impingement syndrome: a cohort study

**DOI:** 10.1186/1471-2474-15-324

**Published:** 2014-09-29

**Authors:** Rahul Bhattacharyya, Kimberley Edwards, Angus W Wallace

**Affiliations:** Trauma and Orthopaedics, West of Scotland deanery, UK; Centre for Sports Medicine, Division of Orthopaedics and Accident Surgery, School of Clinical Sciences, University of Nottingham, Nottingham, NG7 2RD UK; Division of Orthopaedic & Accident Surgery, University of Nottingham, Nottingham University Hospitals, Queen’s Medical Centre Campus, Nottingham, NG7 2UH UK

## Abstract

**Background:**

Health Economists in Denmark have reported poor outcomes and low and delayed return to work for patients treated for Sub-Acromial Impingement syndrome (SAIS) by Arthroscopic Sub-Acromial Decompression (ASAD). In this setting it is important to evaluate outcomes following this commonly performed operation to justify undertaking it on our patients. The purpose of the study was to evaluate the effectiveness of ASAD for patients with SAIS and correlate clinical outcome with rate of return to work.

**Methods:**

Prospective cohort study and retrospective review of data from the Nottingham Shoulder database. Inclusion criteria: Patients diagnosed clinically with SAIS by an experienced shoulder surgeon, who have failed conservative treatment (physiotherapy and sub-acromial injection), undergoing ASAD. Pre-operative and 6-month post-operative Oxford Shoulder Score (OSS) and Constant Score (CS) were compared. The rates of return to pre-operative work and hobbies were also analysed. Statistical analysis was carried out using the Wilcoxon signed rank test.

**Results:**

73 patients with OSS (51 also with CS documentation) were included. The improvement in median OSS between pre-operative (24) and 6-month follow-up (39) was +15 (Z = -6.726, p < 0.0001, T = 6, r = 0.55). The difference in median CS between pre-operative (39) and 6-month follow-up (67) was +28 (Z = -5.435, p < 0.0001,T = 6, r = 0.59). Improvement in median pain score was +5 (7,12, p < 0.0001) median ADL was +5.5 (10.5,16, p < 0.0001) median ROM was +13 (18,31, p < 0.0001) and median strength was +4 (3,7, p < 0.0001). 76% returned to their pre-operative level of work (mean time = 11.5 weeks post surgery). 79% returned to pre-operative hobbies at a mean of 11.8 weeks after surgery.

**Conclusion:**

There is a significant improvement in both subjective and objective outcome 6 months after ASAD in patients with SAIS who have had previous failed conservative treatment. The rate of return to work was good for these patients in contrast to that reported for Danish patients. ASAD is a successful method of treatment for patients with SAIS who have had an initial trial of failed conservative treatment.

## Background

Sub-Acromial Impingement Syndrome (SAIS) is the commonest disorder of the shoulder, accounting for 44-65% of all complaints of shoulder pain [1–3]. It results from an “inflammation and degeneration of the anatomical structures in the region of the sub-acromial space” [4].

Treatment options for SAIS can be classified into conservative or surgical. Conservative management includes exercise therapy, ultrasound treatment and sub-acromial injections. Previous research has shown exercise to be an effective treatment for SAIS [5–9]. Different exercise regimens include supervised exercise, home exercise programs and exercise associated with manual therapy. Exercise improves pain [10–14] and function [12, 15–18], but does not improve shoulder strength [10, 15, 18].

Sub-acromial injections can be used to treat SAIS. A Randomised Controlled Trial (RCT) by Karthikeyan et al. [19] showed that methylprednisolone conferred significant benefits on patients symptoms at six weeks post injection. A RCT by Crawshaw et al. [20] concluded that corticosteroid injections combined with exercise was only successful in achieving short-term benefit.

Arthroscopic and open Sub-Acromial Decompression are the surgical treatment options. Checroun et al. [21] reviewed 34 studies (1,935 patients) between 1970–96. They recommended Arthroscopic Sub-Acromial Decompression (ASAD) as the initial preferred option with open surgery reserved for arthroscopic failures. ASAD allowed earlier rehabilitation although technically it was more demanding. In clinical practice, surgery is usually offered after an initial period of failed conservative treatment. ASAD is one of the most commonly performed procedures in shoulder surgery in the UK. However, in a recent article in Orthopaedics today [22], doubts have been raised over the effectiveness of ASAD. Health Economists in Denmark have reported low and delayed return to work for patients treated for SAIS with ASAD. Their argument is that there are no financial benefits for the government due to the poor rate of return to work. Surgeons argue that patients achieve good pain relief and a high standard of activities of daily living (ADL’s) after ASAD.

A detailed review of the literature suggests that there is no clear benefit of surgery over conservative treatment. Randomised controlled trials by Brox et al. [23] and Haahr et al. [16] comparing exercises with ASAD found that although individually they are successful treatments, ASAD was not superior to specialized exercise program. Systematic reviews by Dorrestijn et al. [24] and Gebremariam et al. [25] also concluded that ASAD was not superior to conservative treatment. In this setting, it is essential to analyse the outcome following ASAD for patients with SAIS to justify bearing the costs and the risks associated with this commonly performed procedure.

The objectives of this study were:

1. To evaluate the outcome of ASAD for patients with SAIS.
2. To determine the rate and time of return to work and hobbies following ASAD in patients with SAIS.

## Methods

This was a prospective cohort study combined with retrospective review of prospectively collected data from the Nottingham shoulder database. Data was collected prospectively from patients who attended the Nottingham Shoulder and Elbow unit for their initial assessment and 6 month follow–up between 01.09.2011 and 01.03.2012. Prospectively collected data recorded in the Nottingham shoulder database from 01.04.2008 to 01.09.2011 has been reviewed and included in our study, if they fulfilled the inclusion criteria. The Nottingham shoulder database is a record of outcome scores for patients undergoing various shoulder procedures. Patients undergoing ASAD have their pre-operative and 6-month follow-up OSS and CS automatically recorded in this database at their routine follow-up appointments. All patients with a clinical diagnosis of SAIS follow the inclusion criteria below before they are offered ASAD. These patients are then prospectively added to the database. Therefore, the data collected prospectively in this study was also added to the database following exactly the same protocol. This ensured that the data was homogenous.

### Inclusion criteria

Patients diagnosed clinically with SAIS by an experienced shoulder surgeon, were included in our study. Patients typically presented with a history of shoulder pain, which worsened with overhead activity. On examination, a painful arc of movement between 60 and 120 degrees of abduction was present and there was tenderness at the insertion of the rotator cuff. Positive impingement tests such as the Neer’s and Hawkins-Kennedy test were also used to make the diagnosis. Plain radiographs were undertaken to exclude alternative causes of shoulder pain such as glenohumeral osteoarthritis. MRI scans were not routinely used unless there was a clinical suspicion of an associated rotator cuff tear. All patients had a trial of conservative therapy in the form of standard physiotherapy and at least one sub-acromial injection of corticosteroid and local anaesthetic by an experienced shoulder surgeon. The minimum duration of symptoms was six months. Those patients who were still significantly symptomatic then underwent an ASAD followed by standard post-operative physiotherapy.

The Arthroscopic Sub -Acromial Decompression operation was carried out with the patient in the beach chair position. A posterior viewing portal was used, initially for a pre-operative diagnostic arthroscopy and then for a subacromial bursoscopy before proceeding to completing the anterior acromioplasty by clearing the subacromial bursa from the acromion and removing approximately 4 to 5 mm of anterior acromion after releasing the coraco-acromial ligament through an antero-lateral portal. At the end of the procedure a cutting block technique was added in approximately 20% of cases.

### Exclusion criteria

These included:

Patients with full thickness rotator cuff tear, requiring a rotator cuff repair, found on arthroscopy.

Patients with long head of biceps tear found during arthroscopy.

Patients with shoulder instability (on arthroscopy).

Alternative pathologies causing similar shoulder pain such as gleno-humeral osteoarthritis, adhesive capsulitis or fracture.

Patients who failed to attend follow-up at six months post surgery.

### Outcome measures

The primary outcome measure was comparison of the OSS and CS pre-operatively and at six-months follow-up. The secondary outcome measure was to evaluate the rate and time of return to work and hobbies after surgery.

### Sample size calculation

A comparison of two means sample size calculation was performed using standard assumptions for α and β (significance level 5% and power of 80% respectively). Data on OSS and CS were obtained from the shoulder database. Using these data, the sample size calculation showed that to be able to measure a change of 5 points in the OSS we needed 35 patients and to measure a 10-point change in CS we needed 51 patients.

### Data collection

Baseline characteristics such as age, sex, pre-morbid level of work and shoulder dominance were recorded. The OSS and CS were calculated by a physiotherapist or research fellow (trained to calculate these scores accurately). The operating surgeon did not obtain these scores from patients to avoid measurement bias. The individual components of the CS: Pain, Activities of Daily Living (ADL), Range Of Motion (ROM) and strength were recorded separately. The pre-operative and six-month follow-up OSS and CS (including the individual components) were then compared to analyze the difference achieved after ASAD.

A 10-point improvement in the CS and 5-point improvement in the OSS at six-months post ASAD was considered to be clinically relevant.

The OSS and CS are validated outcome scores used for shoulder pathologies. They combine both subjective (OSS and pain and ADL of CS) and objective (ROM and strength of CS) assessment of the patient. The OSS [26] assesses various aspects of activities of daily living. It consists of a 12–item questionnaire, which was used where each question scored from 0 (impossible to do) to 4 (no trouble at all). The total score is 48. A higher OSS indicates a better outcome. The CS [27] has 4 individual components. Pain is assessed on an analogue score from 15 (no pain) to 0 (maximum pain). The ADLs (20 points) has four sub sections: Work (4 points), Recreation (2 points), Sleep (2 points) and position (arm position: waist = 2 points, xiphoid = 4 points, neck = 6 points, up to top of head = 8 points, above head = 10 points). ROM (40 points) is assessed for flexion, abduction, external rotation and internal rotation. Finally strength measured using a spring balance contributes 25 points to the CS. This was attached distally to the wrist of the patient. Strength was measured with the arm in 90 degrees of elevation in the plane of the scapula (30 degrees in front of the coronal plane) and with the elbow straight. The palm of the hand was pronated facing the floor. The patient was asked to exert their resisted elevation for 5 seconds. It was repeated on 3 occasions. The average in pound (lb) was noted. The measurement should be pain free. If pain is involved the patient gets 0 points. If the patient was unable to achieve 90 degrees of elevation in the scapula plane then 0 points are given. The higher the CS the better is the outcome.

### Statistical analysis

A statistician who is a part of the research team at the Nottingham Shoulder and Elbow unit was consulted for both the sample size calculation and the analysis of outcome measures. The Wilcoxon signed rank test was used for statistical analysis, as the data was non-parametric.

### Questionnaire part of the study

One of the criticisms by the Danish health economists [22] was the poor and delayed return to work for patients who had surgery for SAIS. In order to analyse this for our cohort of patients, a questionnaire was designed to evaluate the rate and time to return to work and hobbies. We assessed: the pre-operative occupation of the patient, whether they could return to the same job post-operatively, the time to return to work, ability to undertake hobbies after surgery and time to return to hobbies. For retired patients the rate and time of return to their pre-operative level of normal daily activities were calculated. We analyzed the rate of return to work, the time interval from surgery to return to work, rate of return to pre-operative hobbies and the time of return to hobbies after surgery.

### Ethics statement

No formal ethical approval was required for this study. This study was a health service evaluation to evaluate the effectiveness of Arthroscopic Sub-Acromial Decompression (ASAD) for patients with sub-acromial impingement. It is routine practice for the Nottingham Shoulder and Elbow unit to score patients in order to evaluate outcome and this data is stored in the shoulder database. In this study, the prospective Oxford Shoulder score and Constant score we have collected is the same as is routinely done for all patients as part of clinical practice. The study has added to this database and reviewed these outcomes and analysed the data to determine if ASAD is a successful treatment for Sub-Acromial impingement. This was registered and approved by the local Research and Development department but did not require formal approval by the ethical board.

### Consent statement

Outcome scores for patients were calculated routinely as part of evaluation of practice and therefore no formal written consent was necessary for this study.

## Results

92 patients matched the inclusion criteria. 19 patients failed to attend appropriate follow-up and were therefore excluded from the study. Finally, 73 patients were included.

21 patients were prospectively included between 01.09.2011 to 01.03.2012. 52 patients were included from the Nottingham Shoulder database between 01.04.2008 to 01.09.2011 after matching the inclusion criteria.

All 21 prospectively included patients had both the OSS and CS calculated. Amongst the 52 patients included from the database, every patient had a pre-operative and six month follow-up OSS recorded. However, 30/52 patients had pre-operative and six month follow-up CS recorded, including the individual components of the CS (pain, ADL, ROM, strength). Therefore, we had 73 patients with OSS and 51 with CS.

The mean age was 56 years (age range: 24–83 years). 33 were male and 40 were female. 43/73 patients had affected dominant shoulders. Minimum duration of symptoms for all patients was 6 months – Table 1.

**Table 1:** **Baseline characteristics**

Characteristic	Patients with SAIS undergoing ASAD (n = 73)
Mean age (range)	56 (24–83)
Gender (M: F)	33:40
Minimum duration of symptoms	6 months
Side (Dominant: Non Dominant)	43:30
Median OSS at baseline	24

The pre-operative median OSS was 24 (interquartile range: 18–32, mean = 25.12). The six-month follow-up median OSS was 39 (interquartile range 28–44, mean = 35.28). The improvement in median OSS was + 15, mean OSS was 10.16 (Z = -6.726, p < 0.0001, T = 6, r = 0.55).

The pre-operative median CS was 39, mean =41.90, interquartile range 28–54.5. The six month follow-up median CS was 67, mean = 64.54, interquartile range 52.5-79.5. The improvement in median CS was +28; mean CS was 22.64 (Z = -5.435, p < 0.0001,T = 6, r = 0.59).

We analysed the individual components of the CS. Pre-operative pain scores were: median = 7, interquartile range = 4–10, mean = 7.375. Six month follow-up pain scores: median = 12, interquartile range = 8–15, mean = 10.60. The difference between pre-operative and six month follow-up scores was: median = +5, mean = 3.225. (Z = -4.329, p < 0.0001, r = 0.44, T = 8).

Pre-operative ADL scores were: median = 10.5, interquartile range = 6–12, mean = 9.42. Six month follow-up pain scores: median = 16, interquartile range = 10.75-19, mean = 14.81. The difference between pre-operative and six month follow-up scores was: median = +5.5, mean = 5.39. (Z = - -5.435, p < 0.0001, r = 0.55, T = 5).

Pre-operative ROM scores were: median = 18, interquartile range = 13.5-28, mean = 19.125. Six month follow-up pain scores: median = 31, interquartile range = 22–34.5, mean = 29.0625. The difference between pre-operative and six month follow-up scores was: median = +13, mean = 9.9375. (Z = -5.193, p < 0.0001, T = 6, R = 0.52).

Pre-operative strength scores were: median =3, interquartile range = 0–9.5, mean =6. Six month follow-up pain scores: median =7, interquartile range =4-14, mean =9.30. The difference between pre-operative and six month follow up scores was: median = +4, mean = 3.30. (Z = -3.560, p < 0.0001, T = 10, r = 0.36). The results stated above are summarized in Table 2.

**Table 2:** **Summary of outcomes (OSS and CS) 6 months after ASAD**

Score	Baseline (median score)	6 months follow up (median score)	Improvement in median score	Wilcoxon signed rank test
OSS	24	39	+15	Z = -6.726, T = 6, r = 0.55,p < 0.0001
CS	39	67	+28	Z = -5.435, T = 6, r = 0.59, p < 0.0001
Pain	7	12	+5	Z = -4.329, T = 8, r = 0.44, p < 0.0001
ADL	10.5	16	+5.5	Z = - -5.435,T = 5, r = 0.55, p < 0.0001
ROM	18	31	+13	Z = -5.193, T = 6, R = 0.52,p < 0.0001
Strength	3	7	+4	Z = -3.560, T = 10, r = 0.36,p < 0.0001

55/73 (75.3%) patients had a ≥ 5-point improvement in OSS (Clinically relevant). 39/51 (76.5%) patients had a ≥10-point improvement in CS. (Clinically relevant).

### Questionnaire analysis

35 questionnaires (on return to work and hobbies) were returned in the time period of the study. 1 patient had died 3 months after surgery due to unrelated medical complications and hence had to be excluded from the analysis. 27/34 patients were employed at the time of surgery (7/34 - retired) but none were able to work due to the severity of the symptoms. 26/34 (76.5%) patients returned to their pre-operative work. The mean time to return to work was 11.5 weeks. 27/34 (79.4%) patients returned to their hobbies after surgery. The mean time to return to hobbies was 11.8 weeks. Amongst the patients who did not return to their pre-operative work at 6 months post surgery, 2 patients were retired. 7/8 patients had their dominant shoulder operated on. 2/8 patients were manual workers.

## Discussion

The findings from our study have demonstrated significant improvement in outcome for patients with SAIS undergoing ASAD, who have had previous failed conservative treatment with standard physiotherapy and at least one sub-acromial injection. The median OSS improved significantly at 6 months after ASAD. This implies that patients have reported benefits in their activities of daily living. CS is a combination of subjective and objective outcomes. In our study the median CS at six-months follow-up improved by 28 points which was highly statistically and clinically relevant. Furthermore, the improvements in the individual components of the CS highlights excellent pain relief, objective benefits in ROM and also increase in shoulder strength. Any improvement in OSS and CS would be of benefit to the patient. However, experienced shoulder surgeons in our unit believe that a minimum of 5-point improvement in OSS and 10-point improvement in CS would provide maximum clinical benefit. There are no published studies in literature that have investigated this. Our study showed that 75% patients achieved a minimum of 5-point benefit in OSS and 77% patients achieved a minimum of 10-point improvement in CS. This is a significant result and highlights the success of ASAD for patients with SAIS.

The main purpose of this study was to evaluate our practice to justify undertaking ASAD on our patients after the recent doubts raised by the Danish health economists [22]. They have questioned the financial benefits to the government due to the low (rate) and delayed return to work for these patients post surgery and have also reported poor outcomes following this procedure. In contrast, we found excellent subjective and objective outcomes 6 months following ASAD. Furthermore, 77% of our patients returned to work and 79% of patients returned to their pre-operative hobbies at a mean of 3 months after surgery. A recent study [28], evaluated time to return to work for patients (with SAIS) undergoing ASAD. 166 patients were included retrospectively. The time to return to work was 11.1 weeks, which is similar to our findings. A cohort study by McClelland et al. [29] included 68 patients who were clinically diagnosed with SAIS and had failed conservative treatment for a minimum of six months. They found that 74% of the workers returned to their previous work within 3 months. A prospective cohort study by Charalambous et al. [30] evaluated return to work rates in the British population. They included 70 patients. 90% of patients returned to work within 6 weeks. These studies have demonstrated good rate of return to work following ASAD for patients with SAIS.

SAIS is the commonest shoulder pathology [1–3]; however there is no clear consensus from published evidence on the best method of treatment for this condition. Numerous studies have been undertaken comparing treatment modalities for SAIS with various outcome measures and length of follow up. Brox et al. [23] carried out a RCT comparing ASAD and supervised exercise with placebo. They included 125 patients with 2½-year follow-up. The results showed that ASAD and supervised exercises were individually successful treatments when compared to placebo but there was no evidence to support surgery over conservative management. The Neer shoulder score was their main outcome measure. This assessed pain, ROM and clinical testing of muscle strength, stability and radiological measurement but there was no patient reported outcome measure used. This was a drawback of the study. In our study, we have used the OSS, which is a patient reported outcome score and the CS, which combined patient and surgeon reported measures. We have also included return to work data, which is an important outcome measure both for the patient and financially for the government. This RCT and our study have found ASAD to be a successful treatment option for patients with SAIS although the criteria for assessing outcome were different. Another RCT by Haahr et al. [16] compared ASAD with physiotherapy involving training exercises aimed at strengthening of the peri-scapular and the rotator cuff muscles. The inclusion criteria for this study were very similar to our study. Their patients had minimum of 6 months duration of symptoms and had failed initial conservative treatment. The CS and the pain and dysfunction score were used to evaluate outcome. At 1 year follow – up there was no significant difference between surgery and physiotherapy. This study used the CS similar to our study but had no patient reported outcome measure or return to work data.

A prospective cohort study by Patel et al. [31] evaluated the effectiveness of ASAD in patients who had failed conservative treatment. 114 patients were evaluated at a mean of 19 months follow-up (range 12–41 months). Subjective assessment of patient satisfaction and comparison of the abbreviated CS, full CS and time to return to work were undertaken. The OSS was not used in this study. 75% patients were subjectively satisfied with the outcome of their operation. There were significant improvements in the individual parameters of the CS. Strength could not be compared, as it was not measured pre-operatively. The follow-up period was variable in this study, ranging between 12 – 41 months, which could have impacted on the quality of the results. We evaluated patients at 6 months after surgery. This study also did not use a patient reported outcome measure.

A systematic review by Dorrestijn et al. [24] compared the effectiveness of conservative treatment and surgery for patients with SAIS. 323 patients from four RCT’s (two were of medium quality and two of low quality) were included. The main endpoints were reduction in pain and improvement in shoulder function. The analysis showed no difference between conservative management and surgical treatment. A limitation of this review was that only four trials, which were of low and medium quality could be included. Another recent systematic review by Gebremariam et al. [25] aimed to compare surgical and conservative management for SAIS. 5 RCT’s and one systematic review were included. No evidence was found for the superiority of ASAD versus conservative treatment in the short, mid and long term or in favour of one surgical technique when compared with another. However, they recommended conservative treatment due to probability of reduced complication rates and ASAD as the first line surgical option.

The quality of evidence is poor with regards to the best method of treatment for SAIS, as presented above. Furthermore, very few studies have used a combination of validated objective and subjective outcome scores such as the OSS and the CS, which limited the quality of their results.

### Strengths and limitations of our study

Combining patient reported outcome measure (OSS) together with objective outcome (CS) is a major strength of the study. Additionally we have recorded important data on the rate of return to work and hobbies for patients following ASAD. A sample size calculation was performed prior to the study, which ensured adequate numbers of patients were recruited to add strength to the study.

A limitation of our study is the absence of a comparison group. Therefore, we cannot compare ASAD to specialized exercise programs or sub-acromial injections for this cohort of patients.

An area of future research is to compare ASAD with specialized exercise therapy or sub-acromial injection in patients with SAIS using a combination of the OSS and CS together with data on rate of return to work.

## Conclusion

We can conclude that for patients with a clinical diagnosis of SAIS who have had initial failed conservative treatment with standard physiotherapy and at least one sub-acromial space injection of steroid and local anaesthetic (by a shoulder specialist), ASAD is a successful method of treatment. This study adds to the literature because it has shown significant (statistically and clinically) benefit in both subjective and objective outcome by the use of validated outcome scores following ASAD for patients with SAIS.
